# Selections and Abstracts

**Published:** 1903-02

**Authors:** 


### SELECTIONS AND ABSTRACTS.

---

### William Beaumont—A Pioneer American Physiologist.*

Come with me for a few moments on a lovely June day in 1822, to what were then far-off northern wilds, to the island of Michilimacinac, where the waters of Lake Michigan and Lake Huron unite, and where stands Fort Mackinac, rich in the memories of Indian and voyageur, one of the four important posts of the upper lakes in the days when the rose and the fleur-de-lis strove for the mastery of the Western world. Here the noble Marquette labored for his Lord, and here beneath the chapel of St. Ignace they laid his bones to rest. Here the intrepid La Salle, the brave Tonty, and the resolute Du Luht had halted in their wild wanderings. Its palisades and block-houses had echoed the war-whoops of Ojibways and Ottawas, or Hurons and Iroquois, and the old fort had been the scene of bloody massacres and hard-fought fights, but at the conclusion of the War of 1812, after two centuries of struggle, peace settled at last on the island. The fort was occupied by United States troops, who kept the Indians in check and did general police duty on the frontier, and the place had become a rendezvous for Indians and voyageurs in the employ of the American Fur Company. On the bright spring morning the village presented an animated scene. The annual return tide to the trading-post was in full course, and the beach was thronged with canoes and batteaux laden with the pelts of the winter's hunt. Voyageurs and Indians, men, women, and children, with here and there a few soldiers, made up a motley crowd. Suddenly from the company's store there is a loud report of a gun, and amid the confusion and excitement the rumor spreads of an accident, and there is the hurrying of messengers to the barracks for a doctor. In a few minutes (Beaumont says twenty-five or thirty, an eye-witness says three) an elert-looking man in the uniform of a U. S. Army surgeon made

*An address by William Osler, M.D., Baltimore, Professor of Medicine, Johns Hopkins University, before the St. Louis Medical Society, October 4, 1902.

his way through the crowd, and was at the side of a young French Canadian who had been wounded by the discharge of a gun, and with the composure bred of an exceptional experience of such injuries, prepared to make the examination. Though youthful in appearance, Surgeon Beaumont had seen much service, and at the capture of York and at the investment of Plattsburg he had shown a coolness and bravery under fire which had won high praise from his superior officers. The man and the opportunity had met —the outcome is my story of this evening.

### I. THE OPPORTUNITY—ALEXIS ST. MARTIN.

On the morning of June 6th a young French Canadian, Alexis St. Martin, was standing in the company's store, "where one of the party was holding a shotgun (not a musket), which was accidentally discharged, the whole charge entering St. Martin's body. The muzzle was not over three feet from him—I think not more than two. The wadding entered, as well as pieces of his clothing; his shirt took fire; he fell, as we supposed, dead."

"Doctor Beaumont, the surgeon of the fort, was immediately sent for, and reached the wounded man in a very short time, probably three minutes. We had just gotten him on a cot, and were taking off some of his clothing. After the doctor had extracted part of the shot, together with pieces of clothing, and dressed his wound carefully, Robert Stuart and others assisting, he left him, remarking: 'The man cannot live thirty-six hours; I will come and see him by and by.' In two or three hours he visited him again, expressing surprise at finding him doing better than he had anticipated. The next day, after getting out more shot and clothing, and cutting off ragged edges of the wound, he informed Mr. Stuart, in my presence, that he thought he would recover." *

The description of the wound has been so often quoted as reported in Beaumont's work that I give here the interesting summary which I find in a "Memorial" presented to the Senate and House of Representatives by Beaumont. "The wound was re-

*Statement of G. G. Hubbard, an officer of the company, who was present when St. Martin was shot, quoted by Dr. J. R. Bailey, of Mackinac Island, in his address on the occasion of the Beaumont Memorial Exercises, Mackinac Island, July 10, 1900. *The Physician and Surgeon*, December, 1900.

ceived just under the left breast, and supposed, at the time, to have been mortal. A large portion of the side was blown off, the ribs fractured, and openings made into the cavities of the chest and abdomen, through which protruded portions of the lungs and stomach, much lacerated and burnt, exhibiting altogether an appalling and hopeless case. The diaphragm was lacerated and a perforation made directly into the cavity of the stomach, through which food was escaping at the time your memorialist was called to his relief. His life was at first wholly despaired of, but he very unexpectedly survived the immediate effects of the wound, and necessarily continued a long time under the constant professional care and treatment of your memorialist, and, by the blessing of God, finally recovered his health and strength.

"At the end of about ten months the wound was partially healed, but he was still an object altogether miserable and helpless. In this situation he was declared a 'common pauper' by the civil authorities of the county, and it was resolved by them that they were not able nor required to provide for or support, and finally declined taking care of him, and, in pursuance of what they probably believed to be their public duty, authorized by the laws of the territory, were about to transport him, in this condition, to the place of his nativity in Lower Canada, a distance of more than fifteen hundred miles.

"Believing the life of St. Martin must inevitably be sacrificed if such an attempt to remove him should be carried into execution at that time, your memorialist, after earnest, repeated, but unavailing remonstrances against such a course of proceedings, resolved, as the only way to rescue St. Martin from impending misery and death, to arrest the process of transportation and prevent the consequent suffering, by taking him into his own private family, where all the care and attention were bestowed that his condition required.

"St. Martin was, at this time, as before intimated, altogether helpless, and suffering under the debilitating effects of his wounds—naked and destitute of everything. In this situation your memorialist received, kept, nursed, medically and surgically treated and sustained him, at much inconvenience and expense, for nearly two years, dressing his wounds daily, and for considerable part of the time twice a day; nursed him, fed him, clothed him, lodged him, and furnished him with such necessaries and comforts as his condition and suffering required.

"At the end of these two years he had become able to walk and help himself a little, though unable to provide for his own necessities. In this situation your memorialist retained St. Martin in his family for the special purpose of making physiological experiments."

In the month of May, 1825, Beaumont began the experiment. In June he was ordered to Fort Niagara, where, taking the man with him, he continued the experiments until August. He then took him to Burlington and to Plattsburg. From the latter place St. Martin returned to Canada, without obtaining Beaumont's consent. He remained in Canada four years, worked as a voyageur, married, and had two children. In 1829 Beaumont succeeded in getting back to St. Martin, and the American Fur Company engaged him and transported him to Fort Crawford, on the Upper Mississippi. The side and wound were in the same condition as in 1825. Experiments were continued uninterruptedly until March, 1831, when circumstances made it expedient that he should return with his family to Lower Canada. The "circumstances," as we gather from letters, were the discontent and homesickness of his wife. As illustrating the mode of travel, Beaumont states that St. Martin took his family in an open canoe "*via* the Mississippi, passing by St. Louis, ascended the Ohio river, then crossed the State of Ohio to the Lakes, and descended the Erie and Ontario and the River St. Lawrence to Montreal, where they arrived in June." Dr. Beaumont often lays stress on the physical vigor of St. Martin as showing how completely he had recovered from the wound. In November, 1832, he again engaged himself to submit to another series of experiments in Plattsburg and Washington. The last recorded experiment is in November, 1833.

Among the Beaumont papers, for an examination of which I am much indebted to his daughter, Mrs. Keim (Appendix A), there is a large mass of correspondence relating to St. Martin, extending from 1827, two years after he had left the doctor's employ, to October, 1853. Alexis was in Dr. Beaumont's employ in the periods already specified. In 1833 he was enrolled in the United States Army at Washington as Sergeant Alexis St. Martin, of a detachment of orderlies stationed at the War Department. He was then twenty-eight years of age, and was five feet five inches in height.

Among the papers there are two articles of agreement, both signed by the contracting parties, one dated October 19th, 1833, and the other November 7th of the same year. In the former he bound himself for a term of one year, to "serve, abide, and continue with the said William Beaumont, wherever he shall go or travel or reside in any part of the world, his covenant servant, and diligently and faithfully, etc.; . . . that he, the said Alexis, will at all times during said term, when thereto directed or required by said William, submit to assist and promote by all means in his power such philosophical or medical experiments as the said William shall direct or cause to be made on or in the stomach of him, the said Alexis, either through and by means of the aperture or opening thereto in the side of him, the said Alexis, or otherwise, and will obey, suffer, and comply with all reasonable and proper orders of or experiments of the said William in relation thereto and in relation to the exhibiting and showing of his said stomach and the powers and properties thereto and of the appurtenances and the powers, properties and situation and state of the contents thereof." The agreement was that he should be paid his board and lodging and $150 for the year. In the other agreement it is for two years, and the remuneration $400. He was paid a certain amount of the money down.

There are some letters from Alexis himself, all written for him and signed with his mark. In June, 1834, he writes that his wife was not willing to let him go, and thinks that he can do a great deal better to stay at home. From this time on Alexis was never again in Dr. Beaumont's employ.

There is a most interesting and protracted correspondence in the years 1836, 1837, 1838, 1839, 1840, 1842, 1846, 1851 and 1852, all relating to attempts to induce Alexis to come to St. Louis. For the greater part of this time he was in Berthier, in the district of Montreal, and the correspondence was chiefly conducted with a Mr. William Morrison, who had been in the Northwest for trade, and who took the greatest interest in Alexis and tried to induce him to go to St. Louis. (See Appendix B.)

In 1846 Beaumont sent his son Israel for Alexis, and in a letter dated August 9, 1846, his son writes from Troy: "I have just returned from Montreal, but without Alexis. Upon arriving at Berthier I found that he owned and lived on a farm about fifteen miles southwest of the village." Nothing would induce him to go.

The correspondence with Mr. Morrison in 1851 and 1852 is most voluminous, and Dr. Beaumont offered Alexis $500 for the year, with comfortable support for his family. He agreed at one time to go, but it was too late in the winter, and he could not get away.

The last letter of this series is dated October 15, 1852, and is from Dr. Beaumont to Alexis, whom he addresses as *Mon Ami.* Two sentences in this are worth quoting: "Without reference to past efforts and disappointments—or expectation of ever obtaining your services again for the purpose of experiments, etc., upon the proposals and conditions heretofore made and suggested, I now proffer to you in faith and sincerity, new, and I hope satisfactory, terms and conditions to ensure your prompt and faithful compliance with my most fervent desire to have you again with me—not only for my own individual gratification, and the benefits of medical science, but also for your own and family's present good and future welfare." He concludes with: "I can say no more, Alexis—you know what I *have* done for you many years since—what I have been *trying*, and am still anxious and wishing to do with and for you—what efforts, anxieties, anticipations and disappointments I have suffered from your non-fulfillment of my expectations. Don't disappoint me more nor forfeit the bounties and blessings reserved for you."

So much interest was excited by the report of the experiments that it was suggested to Beaumont that he should take Alexis to Europe and submit him there to a more extended series of observations by skilled physiologists. Writing June 10, 1833, he says: "I shall engage him for five or six years if he will agree, of which I expect there is no doubt. He has always been pleased with the idea of going to France. I feel much gratified at the expression of Mr. Livingstone's desire that we should visit Paris, and shall duly consider the interest he takes in the subject and make the best arrangements I can to meet his views and yours." Mr. Livingstone, the American minister, wrote from Paris March 18, 1834, saying that he had submitted the work to Orfila and the Academy of Science, which had appointed a committee to determine if additional experiments were necessary, and whether it was advisable to send to America for Alexis. Nothing, I believe, ever came of this, nor, so far as I can find, did Alexis visit Paris. Other attempts were made to secure him for purposes of study. In 1840 a student of Dr. Beaumont's, George Johnson, then at the University of Pennsylvania, wrote saying that Dr. Jackson had told him of efforts made to get Alexis to London, and Dr. Gibson informed him that the Medical Society of London had raised £300 or £400 to induce St. Martin to come, and that he, Dr. Gibson, had been trying to find St. Martin for his London friends. There are letters in the same year from Dr. R. D. Thomson, of London, to Professor Silliman urging him to arrange that Dr. Beaumont and Alexis should visit London. In 1856 St. Martin was under the observation of Dr. Francis Gurney Smith, in Philadelphia, who reported a brief series of experiments, so far I as know, the only other report made on him.*

St. Martin had to stand a good deal of chafing about the hole in his side. His comrades called him "the man with a lid on his stomach." In his memorial address Mr. C. S. Osborne, of Sault Ste. Marie, states that Miss Catherwood tells a story of Etienne St. Martin fighting with Charlie Charette because Charlie ridiculed his brother. Etienne stabbed him severely, and swore that he would kill the whole brigade if they did not stop deriding his brother's stomach.

At one time St. Martin traveled about exhibiting the wound to physicians, medical students, and before medical societies. In a copy of Beaumont's work, formerly belonging to Austin Flint, Jr., and now in the possession of a physician of St. Louis, there is a photograph of Alexis sent to Dr. Flint. There are statements made that he went to Europe, but of such a visit I can find no record.

*Medical Examiner, 1856, and Experiments on Digestion, Philadelphia, 1856.

My interest in St. Martin was of quite the general character of a teacher of physiology, who every session referred to his remarkable wound, and showed Beaumont's book with the illustration. In the spring of 1880, while still a resident of Montreal, I saw a notice in the newspapers of his death at St. Thomas. I immediately wrote to a physician and to the parish priest, urging them to secure me the privilege of an autopsy and offering to pay a fair sum for the stomach, which I agreed to place in the Army Medical Museum at Washington, but without avail. Subsequently, through the kindness of the Hon. Mr. Justice Baby, I obtained the following details of St. Martin's later life, and the picture here given, which was taken a year before his death so as to show the wound, which I here show you. Judge Baby writes to his friend, Prof. D. C. MacCallum, of Montreal, as follows: "I have much pleasure to-day in placing in your hands such information about St. Martin as Rev. Mr. Chicoine, Curé of St. Thomas, has just handed over to me. Alexis Bidigan, *dit* St. Martin, died at St. Thomas de Joliette on the 24th of June, 1880, and was buried in the cemetery of the parish on the 28th of the same month. The last sacraments of the Catholic Church were ministered to him by the Rev. Curé Chicoine, who also attended at his burial service. The body was then in such an advanced stage of decomposition that it could not be admitted into the church, but had to be left outside during the funeral service. The family resisted all requests—most pressing as they were—on the part of the members of the medical profession for an autopsy, and also kept the body at home much longer than usual, and during a hot spell of weather, so as to allow decomposition to set in and baffle, as they thought, the doctors of the surrounding country and others. They had also the grave dug eight feet below the surface of the ground in order to prevent any attempt at a resurrection. When he died St. Martin was eighty-three years of age, and left a widow, whose maiden name was Marie Joly. She survived him by nearly seven years, dying at St. Thomas on the 20th of April, 1887, at the very old age of ninety years. They left four children, still alive—Alexis, Charles, Henriette, and Marie.

"Now I add the following details for myself. When I came to know St. Martin it must have been a few years before his death. A lawsuit brought him to my office here in Joliette. I was seized with his interests; he came to my office a good many times, during which visits he spoke to me at great length of his former life, how his wound had been caused, his peregrinations through Europe and the United States, etc. He showed me his wound. He complained bitterly of some doctors who had awfully misused him, and had kind words for others. He had made considerable money during his tours, but had expended and thrown it all away in a frolicksome way, especially in the old country. When I came across him he was rather poor, living on a small, scanty farm in St. Thomas, and very much addicted to drink, almost a drunkard, one might say. He was a tall, lean man, with a very dark complexion, and appeared to me then of a morose disposition.

II. THE BOOK.

In the four periods in which Alexis had been under the care and study of Beaumont a large series of observations had been recorded, amounting in all to 238. A preliminary account of the case and of the first group of observations appeared in the *Philadelphia Medical Recorder* in January, 1825. During the stay in Washington in 1832 the great importance of the observations had become impressed on the Sugeon-General, Dr. Lovell, who seems to have acted in a most generous and kindly spirit. Beaumont tried to induce him to undertake the arrangement of the observations, but Lovell insisted that he should do the work himself. In the spring of 1833 Alexis was taken to New York and there shown to the prominent members of the profession, and careful drawings and colored sketches were made of the wound by Mr. King. A prospectus of the work was issued and was distributed by the Surgeon-General, who speaks in a letter of sending them to Dr. Franklin Bache and to Dr. Stewart of Philadelphia, and in a letter from Dr. Bache to Dr. Beaumont acknowledging the receipt of a bottle of gastric juice, Bache states that he has placed the prospectus in Mr. Judah Dobson's store and has asked for subscribers. Beaumont did not find New York a very congenial place. He complained of the difficulty of doing the work owing to the vexations of social intercourse. He applied for permission to go to Plattsburg, in order to complete the book. After having made inquiries in New York and Philadelphia about terms of publication, he decided, as the work had to be issued at his own expense, that it could be as well and much more cheaply printed at Plattsburg, where he would also have the advice and help of his cousin, Dr. Samuel Beaumont. In a letter to the Surgeon-General, dated June 10, 1833, he acknowledges the permission to go to Plattsburg, and says: "I shall make my arrangements to leave here for Pl. in about a week to *rush* the execution of the book as fast as possible. I am now having the drawings taken by Mr. King engraved here."

The summer was occupied in making a fresh series of experiments and getting the work in type. On December 3 he writes the Surgeon-General that the book will be ready for distribution in a few days, and that 1,000 copies will be printed.

The work is an octavo volume of 280 pages, entitled: "Experiments and Observations on the Gastric Juice and the Physiology of Digestion," by William Beaumont, M.D., Surgeon in the United States Army. Plattsburg. Printed by F. P. Allen, 1833. While it is well and carefully printed, the paper and type are not of the best, and one cannot but regret that Beaumont did not take the advice of Dr. Franklin Bache, who urged him strongly not to have the work printed at Plattsburg, but in Philadelphia, where it could be done in very much better style. The dedication of the work to Joseph Lovell, M.D., Surgeon-General of the United States Army, acknowledges in somewhat laudatory terms the debt which Beaumont felt he owed to his chief, who very gratefully acknowledges the compliment and the kindly feeling, but characterizes the dedication as "somewhat apocryphal."

The work is divided into two main portions; first, the preliminary observations on the general physiology of digestion in seven sections: Section I., of Ailment; Section II., of Hunger and Thirst; Section III., of Satisfaction and Satiety; Section IV., of Mastication, Insalivation and Deglutition; Section V., of Digestion by the Gastric Juice; Section VI., of the Appearance of the Villous Coat, and of the Motions of the Stomach; Section VII., of Chylification and uses of the Bile and Pancreatic Juice. The greater part of the book is occupied by the larger section of the detailed account of the four series of experiments and observations. The work concludes with a series of fifty-one inferences from the foregoing experiments and observations.

The subsequent history of the book itself is of interest, and may be dealt with here. In 1834, copies of the Plattsburg edition, printed by F. P. Allen, were issued by Lilly, Wait & Co., of Boston.

In the Beaumont correspondence there are many letters from a Dr. McCall, in Utica, N. Y., who was an intimate friend of a Mr. Wm. Combe, a brother of the well-known physiologist and popular writer, Dr. Andrew Combe, of Edinburgh. Doubtless it was through this connection that in 1838 Dr. Combe issued an edition in Scotland, with numerous notes and comments. (Appendix C.)

The second edition was issued from Burlington, Vt., in 1847, with the same title-page, but after Second Edition there are the words, "Corrected by Samuel Beaumont, M.D., who was Dr. William Beaumont's cousin." In the preface of this edition, the statement is made that the first edition, though a large one of 3,000 copies, had been exhausted. This does not agree with the statement made in a letter of December 3, 1833, to the Surgeon-General, stating that the edition was to be 1,000 copies. Of course more may have been printed before the type was distributed. While it is stated to be a new and improved edition, so far as I can gather it is a verbatim reprint, with no additional observations, but with a good many minor corrections. In an appendix (D) I give an interesting letter from Dr. Samuel Beaumont with reference to the issue of this edition.

A German edition was issued in 1834 with the following title: "Neue Versuche und Beobachtungen ueber den Magensaft und die Physiologie der Verdauung, auf eine hochst merkwurdige Weise wahrend einer Reihe von 7 Jahren, an einen und demselben Subject angestellt." Beaumont's earlier paper, already referred to, was abstracted in the Magazin der auslandischen Litteratur der gesammten Heilkunde, Hamburg, 1826, and also in the Archives generales de Medecine, Paris, 1828. I cannot find that there was a French edition of the work.

The "Experiments and Observations" attracted universal attention, both at home and abroad. The journals of the period contained very full accounts of the work, and within a few years the valuable editions to our knowledge filtered into the text-books of physiology, which to-day in certain descriptions of the gastric juice and of the phenomena of digestion even the very language of the work is copied.

III. THE VALUE OF BEAUMONT'S OBSERVATIONS.

There had been other instances of artificial gastric fistula in man which had been made the subject of experimental study, but the case of St. Martin stands out from all others on account of the ability and care with which the experiments were conducted. As Dr. Combe says, the value of these experiments consists partly in the admirable opportunities for observation which Beaumont enjoyed, and partly in the candid and truth-seeking spirit in which all his inquiries seem to have been conducted. "It would be difficult to point out any observer who excels him in devotion to truth and freedom from the trammels of theory or prejudice. He tells plainly what he saw and leaves every one to draw his own inferences, or where he lays down conclusions he does so with a degree of modesty and fairness of which few perhaps in his circumstances would have been capable."

To appreciate the value of Beaumont's studies it is necessary to refer for a few moments to our knowledge of the physiology of digestion in the year 1832, the date of the publication. Take, for example, "The Work on Human Physiology" (published in the very year of the appearance of Beaumont's book), by Dunglison, a man of wide learning and thoroughly informed in the literatnre of the subject. The five or six old theories of stomach digestion, concoction, putrefaction, trituration, fermentation, and maceration, are all discussed, and Wm. Hunter's pithy remark is quoted: "Some physiologists will have it that the stomach is a mill, others that it is a fermenting vat, others, again, that it is a stewpan; but in my view of the matter, it is neither a mill, a fermenting vat nor a stew-pan; but a stomach, gentlemen, a stomach."

The theory of chemical solution is accepted. This had been placed on a sound basis by the experiments of Reaumur, Spallanzani, and Stevens, while the studies of Tiedemann and Gmelin and of Prout had done much to solve the problems of the chemistry of the juice. But very much uncertainty existed as to the phenomena occurring during digestion in the stomach, the precise mode of action of the juice, the nature of the juice itself, and its action outside the body. On all these points the observations of Beaumont brought clearness and light where there had been previously the greatest obscurity.

The following may be regarded as the most important of the results of Beaumont's observations: First, the accuracy and completeness of description of the gastric juice itself. You will all recognize the following quotation which has entered into the textbooks and passes current to-day: "Pure gastric juice, when taken directly out of the stomach of a healthy adult, unmixed with any other fluid, save a portion of the mucus of the stomach with which it is most commonly and perhaps always combined, is a clear, transparent fluid; inodorous; a little saltish, and very perceptibly acid. Its taste, when applied to the tongue, is similar to this mucilaginous water slightly acidulated with muriatic acid. It is readily diffusible in water, wine, or spirits; slightly effervesces with alkalies; and is an effectual solvent of the *materia alimentaria.* It possesses the property of coagulating albumen in an eminent degree; is powerfully antiseptic, checking the putrefaction of meat, and effectually restorative of healthy action, when applied to old, fetid sores, and foul, ulcerating surfaces."

Secondly, the confirmation of the observation of Prout that the important acid of the gastric juice was the muriatic or hydrochloric. An analysis of St. Martin's gastric juice was made by Dunglison, at that time a professor in the University of Virginia, and by Benjamin Silliman, of Yale, both of whom determined the presence of free hydrochloric acid. A specimen was sent to the distinguished Swedish chemist, Berzelius, whose report did not arrive in time to be included in the work. In a letter dated July

19, 1834, he writes to Professor Silliman that he had not been able to make a satisfactory analysis of the juice. The letter is published in *Silliman's Journal,* Vol. 27, July, 1835.

Thirdly, the recognition of the fact that the essential elements of the gastric juice and the mucus were separate secretions.

Fourthly, the establishment by direct observation of the profound influence on the secretion of the gastric juice and on digestion of mental disturbances.

Fifthly, a more accurate and fuller comparative study of the digestion in the stomach with digestion outside the body, confirming in a most elaborate series of experiments the older observations of Spallanzani and Stevens.

Sixthly, the refutation of many erroneous opinions relating to gastric digestion and the establishment of a number of minor points of great importance, such as, for instance, the rapid disappearance of water from the stomach through the pylorus, a point brought out by recent experiments, but insisted on and amply proven by Beaumont.

Seventhly, the first comprehensive and thorough study of the motions of the stomach, observations on which, indeed, are based the most of our present knowledge.

And lastly, a study of the digestibility of different articles of diet in the stomach, which remains to-day one of the most important contributions ever made to practical dietetics.

The greater rapidity with which solid food is digested, the injurious effects on the stomach of tea and coffee, when taken in excess, the pernicious influence of alcoholic drinks on the digestion, are constantly referred to. An all-important practical point insisted on by Beaumont needs emphatic reiteration to this generation: "The system requires much less than is generally supplied to it. The stomach disposes of a definite quantity. If more be taken than the actual wants of the economy require, the residue remains in the stomach and becomes a source of irritation and produces a consequent aberration of function, or passes into the lower bowels in an undigested state, and extends to them its deleterious influence. Dyspepsia is oftener the effect of overeating and overdrinking than of any other cause."

One is much impressed, too, in going over the experiments, to note with what modesty Beaumont refers to his own work. He speaks of himself as a humble "inquirer after truth, and a simple experimenter." "Honest objections, no doubt, are entertained against the doctrine of digestion by the gastric juice. That they are so entertained by these gentlemen I have no doubt. And I cheerfully concede to them the merit of great ingenuity, talent and learning in raising objections to the commonly received hypothesis, as well as ability in maintaining their peculiar opinions. But we ought not to allow ourselves to be seduced by the ingenuity of arguments or the blandishments of style. Truth, like beauty, when 'unadorned is adorned the most'; and in prosecuting these experiments and inquiries, I believe I have been guided by its light. Facts are more persuasive than arguments, however ingeniously made, and by their eloquence I hope I have been able to plead for the support and maintenance of those doctrines which have had for their advocates such men as Sydenham, Hunter, Spallanzani, Richerand, Abernethy, Broussais, Philip, Paris, Bostock, the Heidleburg and Paris professors, Dunglison, and a host of other luminaries in the science of physiology."

In reality Beaumont anticipated some of the most recent studies in the physiology of digestion. Doubtless many of you have heard of Professor Pawlow's, of St. Petersburg, new work on the subject. It has been translated into German, and I see that an English edition is advertised. He has studied the gastric juice in an isolated pouch, ingeniously made at the fundus of the stomach of a dog, from which the juice could be obtained in a pure state. One of his results is the very first announced by Beaumont, and confirmed by scores of observations on St. Martin, viz., that, as he says, "the gastric juice never appears to be accumulated in the cavity of the stomach while fasting." Pawlow has shown very clearly that there is a relation between the amount of food taken and the quantity of gastric juice secreted. Beaumont came to the same conclusion: "When aliment is received the juice is given in exact proportion to its requirements for solution." A third point on which Pawlow lays stress is the curve of secretiou of the gastric juice, the manner in which it is poured out during digestion. The greatest secretion, he has shown, takes place in the earlier hours. On this point hear Beaumont: "It (the gastric juice) then begins to exude from the proper vessels, and increases in proportion to the quantity of aliment naturally required and received." And again: "When a due and moderate supply of food has been received it is probable that the whole quantity of gastric juice for its complete solution is secreted and mixed with it in a short time." A fourth point, worked out carefully by Pawlow, is the adaptation of the juice to the nature of the food, on which I do not see any reference by Beaumont, but there are no experiments more full than those in which he deals with the influence of exercise, weather, and the emotions on the quantity of the juice secreted.

### IV. MAN AND DOCTOR.

Sketches of Dr. Beaumont's life have appeared from time to time. There is a worthy memoir by Dr. T. Reyburn in the *St. Louis Medical and Surgical Journal,* 1854, and Dr. A. J. Steele, at the first annual commencement of the Beaumont Medical College, 1887, told well and graphically the story of his life. A few years ago Dr. Frank J. Lutz, of this city, sketched his life for the memorial meeting of the Michigan State Medical Society, on the occasion of the dedication of a Beaumont monument.

Among the papers kindly sent to me by his daughter, Mrs. Keim, are many autobiographical materials, particularly relating to his early studies and to his work as a surgeon in the War of 1812. There is an excellent paper in the handwriting, it is said, of his son, giving a summary of the earlier period of his life. So far as I know this has not been published, and I give it in full:

Dr. William Beaumont was born in the town of Lebanon, Conn., on the 21st day of November, A.D. 1785. His father was a thriving farmer and an active politician of the proud old Jeffersonian school, whose highest boast was his firm support and strict adherence to the honest principles he advocated. William was his third son, who, in the winter of 1806–7, in the twenty-second year of his age, prompted by a spirit of independence and adventure, left the parental roof to seek a fortune and a name. His outfit consisted of a horse and cutter, a barrel of cider, and one hundred dollars of hard-earned money. With this he started, laying his course northwardly, without any particular destination—Honor his rule of action, Truth his only landmark, and trust placed implicitly in Heaven. Traversing the western part of Massachusetts and Vermont in the spring of 1807, he arrived at the little village of Champlain, N. Y., on the Canada frontier—an utter stranger, friendless and alone. But honesty of purpose and true energy invariably work good results. He soon gained the people's confidence and was entrusted with their village school, which he conducted about three years, devoting his leisure hours to the study of medical works from the library of Dr. Seth Pomeroy, his first patron. He then went over to St. Albans, Vt., where he entered the office of Dr. Benjamin Chandler and commenced a regular course of medical reading, which he followed for two years, gaining the utmost confidence and esteem of his kind preceptor and friends. About this time the War of 1812 commenced, and he applied for an appointment in the United States Army successfully. He was appointed assistant surgeon to the Sixth Infantry and joined his regiment at Plattsburg, N. Y., on the 13th of September, 1812. On the 19th of March, 1813, he marched from Plattsburg with the First Brigade, for Sackett's Harbor, where they arrived on the 27th inst. Here he remained in camp till the 22d of April, when he embarked with the troops on Lake Ontario. His journal will best tell this portion of his history:

"April 22d, 1813.—Embarked with Captain Humphreys, Walworth, and Muhlenburg, and companies on board the schooner Julia. The rest of the brigade, and the Second, with Foresith's Rifle Regiment and the Eighth Artillery, on board a ship, brig and schooner—remain in the harbor till next morning.

"23d (11 o'clock a.m.).—Weighs anchor and put out under the impression we were going to Kingston. Got out fifteen or twenty miles—encountered a storm—wind ahead, and the fleet returned to harbor.

"24th (6 o'clock a.m.).—Put sail with a fair wind—mild and pleasant—the fleet sailing in fine order.

"26th.—Wind pretty strong—increasing—waves run high, tossing our vessels roughly. At half-past 4 pass the mouth of Niagara river. This circumstance baffles imagination as to where we are going—first impressed with the idea of Kingston—then to Niagara—but now our destination must be 'Little York.' At sunset came in view of York Town and the Fort, where we lay off some three or four leagues for the night.

"27th.—Sailed into harbor and came to anchor a little below the British garrison. Filled the boats and effected a landing, though not without difficulty and the loss of some men. The British marched their troops down the beach to cut us off at landing, and, though they had every advantage, they could not effect their design. A hot engagement ensued, in which the enemy lost nearly a third of their men and were soon compelled to quit the field, leaving their dead and wounded strewn in every direction. They retired to the garrison, but from the loss sustained in the engagement, the undaunted courage of our men, and the brisk firing from our fleet, with the 12 and 32-pounders, they were soon obliged to evacuate it and retreat with all possible speed. Driven to this alternative, they devised the inhuman project of blowing up their magazine, containing 300 pounds of powder, the explosion of which had well-nigh destroyed our army. Over 300 were wounded and about 60 killed on the spot by stones of all demensions falling, like a shower of hail, in the midst of our ranks. A most distressing scene ensues in the hospital. Nothing is heard but the agonizing groans and supplications of the wounded and the dying. The surgeons wade in blood, cutting off arms and legs and trepanning heads, while the poor sufferers cry, 'O my God! Doctor, relieve me from this misery! I cannot live!' 'Twas enough to touch the veriest heart of steel and move the most relentless savage. Imagine the shocking scene, where fellow-beings lie mashed and mangled—legs and arms broken and sundered—heads and bodies bruised and mutilated to disfigurement! My deepest sympathies were aroused—I cut and slashed for thirty-six hours without food or sleep.

"29th.—Dressed upwards of fifty patients—from simple contusions to the worst of compound fractures—more than half the latter. Performed two cases of amputation and one of trepanning. At 12 p. m. retired to rest my fatigued body and mind."

One month after the taking of York he witnessed the storming of Fort George. The troops were transported from York to "Four-Mile Creek" (in the vicinity of Fort George), where they encamped from the 10th of May to the 27th, when they advanced to the attack. His journal runs thus:

"May 27 (1813).—Embarked at break of day—Col. Scott with 800 men, for the advanced guard, supported by the First Brigade, commanded by General Boyd, moved in concert with the shipping to the enemy's shore and landed under their battery and in front of their fire with surprising success, not losing more than thirty men in the engagement, though the enemy's whole force was placed in the most advantageous situation possible. We routed them from their chosen spot—drove them from the country, and took possession of the town and garrison."

On the 11th of September, 1814, he was at the Battle of Plattsburg, still serving as assistant surgeon, though doing all the duty of a full surgeon. At the close of the war, in 1815, when the army was cut down, he was retained in service, but resigned soon after, deeming himself unjustly treated by the government in having others, younger and less experienced, promoted over him.

In 1816 he settled in Plattsburg and remained there four years in successful practice. In the meantime, his army friends had persuaded him to join the service again, and, having applied, he was reappointed in 1820 and ordered to Fort Mackinac as post surgeon. At the end of the first year he obtained leave of absence, returned to Plattsburg and married one of the most amiable and interesting ladies of that place. (She still survives her honored husband, and in her green old age is loved devotedly by all who know her.) He returned to Mackinac the same year, and in 1822 came in possession of Alexis St. Martin, the subject of his "Experiments on the Gastric Juice." By the accidental discharge of his gun, while hunting, St. Martin had dangerously wounded himself in the abdomen and came under the treatment of Dr. Beaumont, who healed the wound (in itself a triumph of skill almost unequalled), and in 1825 commenced a series of experiments, the results of which have a world-wide publication. These experiments were continued, with various interruptions, for eight years, during which time he was ordered from post to post—now at Niagara, N. Y., anon at Green Bay, Mich., and finally at Fort Crawford, on the Mississippi. in 1834 he was ordered to St. Louis, where he remained in service till 1839, when he resigned. He then commenced service with the citizens of St. Louis, and from that time till the period of his last illness, enjoyed an extensive and distinguished practice, interrupted only by the base attacks of a few disgraceful and malicious knaves (self-deemed members of the medical profession), who sought to destroy a reputation which they could not share. They gained nothing except some little unenviable notoriety, and they have skulked away like famished wolves, to die in their hiding-places.

The dates of Beaumont's commissions in the army are as follows: Surgeon's Mate, Sixth Regiment of Infantry, December 2, 1812; Cavalry, March 27, 1819; Post Surgeon, December 4, 1819; Surgeon First Regiment and Surgeon, November 6, 1826.

From the biographical sketches of Reyburn, Steele and Lutz, and from the personal reminiscences of his friends, Drs. J. B. Johnson, S. Pollock and Wm. McPheeters, who fortunately remains with you, full of years and honors, we gather a clearly defined picture of the latter years of his life. It is that of a faithful, honest, hard-working practitioner, doing his duty to his patients and working with zeal and ability for the best interests of the profession. The strong common sense which he exhibited in his experimental work made him a good physician and a trusty adviser in cases of surgery. Among his letters there are some interesting pictures of his life, particularly in his letters to his cousin, Dr. Samuel Beaumont. Writing him on April 4, 1846, he says:

I have a laborious, lucrative and increasing practice; more than I can possibly attend to, though I have an assistant, Dr. Johnson, a young man who was a pupil of mine from 1835 to 1840. He then went to Philadelphia a year or two to attend lectures and graduated and returned here again in 1842, and has been very busy ever since and is so now, but notwithstanding I decline more practice daily than half the doctors in the city get in a week. You thought when you were here before that there was too much competition for you ever to think of succeeding in business here—

there is ten times as much now and the better I succeed and prosper for it. You must come with a different feeling from your former—with a determination to follow in my wake and stem the current that I will break for you. I am now in the grand climacteric of life, three-score years and over, with equal or more zeal and ability to do good and contribute to professional service than at forty-five, and I now look forward with pleasing anticipation of success and greater usefulness—have ample competence for ourselves and children, and no doleful or dreaded aspect of the future—to be sure I have to wrestle with some adverse circumstances of life, and more particularly to defend myself against the envious, mean and professional jealousies and the consequent prejudices of some men, but I triumph over them all and go ahead in defiance of them.*

His professional work increased enormously with the rapid growth of the city, but he felt, even in his old age, that delicious exhilaration which it is your pleasure and privilege to enjoy here in the West in a degree rarely experienced by our Eastern confrères. Here is a cheery paragraph from a letter dated October 28, 1852: "Domestic affairs are easy, peaceable and pleasant. Health of community good—no severe epidemic diseases prevalent—weather remarkably pleasant—business of all kinds increasing—product of the earth abundant—money plenty—railroads progressing with almost telegraphic speed—I expect to come to Plattsburg next summer, all the way by rail."

But work was becoming more burdensome to a man nearing three score years and ten, and he expresses it in another letter when he says: "There is an immense professional practice in this city. I get tired of it and have been trying hard to withdraw from it altogether, but the more I try the tighter I seem to be held to it by the people. I am actually persecuted, worried and almost worn out with valetudinarian importunities and hypochondriacal groans, repinings and lamentations.—Amen."

He continued at work until March, 1853, when he had an acci-

*He had evidently hopes that when his cousin and son arrived with Alexis they would arrange and plan for another series of experiments, and in another year or two make another book, better than the old one.

dent—a fall while descending some steps. A few weeks later a carbuncle appeared on the neck and proved fatal April 25. One who knew him well wrote the following estimate (quoted by Dr. F. J. Lutz in his sketch of Beaumont):

"He was gifted with strong natural powers, which, working upon an extensive experience in life, resulted in a species of natural sagacity, which, as I suppose, was something peculiar in him and not to be attained by any course of study. His temperament was ardent, but never got the better of his instructed and disciplined judgment, and whenever or however employed, he ever adopted the most judicious means for attaining ends that were always honorable. In the sick-room he was a model of patience and kindness; his intuitive perceptions, guiding a pure benevolence, never failed to inspire confidence, and thus he belonged to that class of physicians whose very presence affords Nature a sensible relief."

You do well, citizens of St. Louis and members of our profession, to cherish the memory of William Beaumont. Alive, you honored and rewarded him, and there is no reproach against you of neglected merit and talents unrecognized. The profession of the northern part of the State of Michigan has honored itself in erecting a monument to his memory near the scene of his disinterested labors in the cause of humanity and science. His name is linked with one of your educational institutions and joined with that of a distinguished laborer in another field of practice. But he has a far higher honor than any you can give him here—the honor that can only come when the man and the opportunity meet and match. Beaumont is the pioneer physiologist of this country, the first to make an important and enduring contribution to this science. His work remains a model of patient, persevering investigation, experiment and research, and the highest praise we can give him is to say that he lived up to and fulfilled the ideals with which he set out and which he expressed when he said: "Truth, like beauty, when 'unadorned, is adorned the most,' and in prosecuting these experiments and inquiries I believe I have been guided by its light."

### APPENDIX A.

The Beaumont papers in the possession of his daughter, Mrs. Keim, of St. Louis, consists of (1) interesting certificates from his preceptors, Drs. Pomeroy and Dr. Chandler, the license from the Third Medical Society of Vermont, the commissions in the U. S. Army, several certificates of honorary membership in societies and the parchment of the M.D. degree conferred upon him, *honoris causa*, by the Columbian University of Washington, 1833; (2) a journal containing his experiences in the War of 1812, from which I give an extract, a journal of his trip to Fort Mackinac; a journal containing the reports of many cases, among them that of St. Martin (in addition there is a protocol of the case in loose folio sheets); a journal of the experiments and a commonplace book of receipts and jottings; (3) an extensive correspondence relating to St. Martin and the book, and many rough drafts of sections of the book; (4) a large mass of personal correspondence, much of it of interest as relating to conditions of practice in St. Louis.

The picture reproduced here in his army uniform is from a miniature; the picture which has been previously reproduced is of an older man from a daguerreotype. It is sasisfactory to know that the ultimate destination of this most valuable collection of papers is the Surgeon-General's Library of the United States Army, of which Dr. Beaumont was so distinguished an ornament.

### APPENDIX B.

On October 20, 1854, he writes to his cousin, Dr. Samuel Beaumont, on the subject of "that old fistulous Alexis," as he calls him. "Alexis' answer to yours is the very facsimile or stereotype of all his Jesuitical letters to me for the last fifteen years. His object seems only to be to get a heavy bonus and undue advance from me, and then disappoint and deceive me, or to palm and impose himself and whole family upon me for support for life.

"I have evaded his designs so far, but I verily fear that the strong and increasing impulse of conscious conviction of the great benefits and important usefulness of further and more accurate physiological investigation of the subject will compel me to still further efforts and sacrifices to obtain him. Physiological authors and most able writers on dietetics and gastric functions generally demand it of me in trumpet tones.

"I must have him at all hazards and obtain the necessary assistance to my individual and private efforts, or transfer him to some competent scientific institution for thorough investigation and report—I must retrieve my past ignorance, imbecility and professional remissness of a quarter of a century or more by double diligence, intense study and untiring application of soul and body to the subject before I die:

Should posthumous Time retain my name,
Let historic truths declare my fame.

"Simultaneous with this I write to Mr. Morrison and Alexis my last and final letters—perhaps, proposing to *him*, as bribe to his cupidity, to give him $500 to come to me *without* his family, for one year—$300 of them for his salary and $200 for the support and contentment of his family to remain in Canada in the meantime—with the privilege of bringing them on here another year, upon my former proposition of $300 a year, at his own expense and responsibility, and support them himself after they get here out of his $300 salary—I think he will take the bait and come on this fall, and when I get him alone again into my keeping and engagement, I will take good care to control him as I please."

APPENDIX C.

Letter from Dr. Andrew Combe, May 1, 1838:

"My Dear Sir—May I beg your acceptance of the accompanying volumes as a small expression of my respect for your character and scientific labors. I need not detain you by repeating in this note the high estimation in which I hold you. The volumes herewith sent will, I trust, convince you of the fact, and that it will not be my fault if you do not receive the credit justly due to your valuable and disinterested services.

"I remain, my dear sir, very respectfully yours,

"ANDW. COMBE."

APPENDIX D.

Letter from Dr. Samuel Beaumont, March 6, 1846:

"Your letter of the 1st of February arrived here in the course of mail, and I have attended to the business which you authorized me to do. I am afraid, however, that you will be disappointed, and perhaps dissatisfied with the arrangement. Mr. Goodrich came here five or six days after I received your letter, and made his proposal, which was to give you every tenth copy for the privilege of publishing an edition. The number proposed to publish was fifteen hundred, which would give you one hundred and fifty copies. I did not like to close the bargain on this condition, and he was not disposed to give any more. This was in the evening. I told him to give me time till the next morning, and I would make up my mind. In the morning, after consultation, I concluded to offer him the copyright for the unexpired time (only one year) for two hundred copies. After some demurring, we closed the bargain. I then thought, and I think still, it was not enough; but it was all I could get. In making up my mind the following considerations presented themselves: First, that the copyright would expire in one year, and he would then have the right to print it without consulting the author; second, that it would be somewhat mortifying to the author not to have his work republished, even if no greater pecuniary benefit was to be obtained by such a republication; and it appeared to me to be quite certain that a new edition would not be soon printed if I let this opportunity slip; third, I have been long anxious, as I presume you have been, to see the work gotten up in a better dress than it originally had, and in a way which will give it a general credit and more notoriety among all classes of the reading public than it has heretofore possessed, in fact make it a standard work; fourth, it has given us a chance to give it a thorough correction, a thing which was very desirable. The work, you recollect, was got up in a great hurry, and a great many errors escaped our notice. You may also recollect that the Philadelphia reviewer spoke of the inaccuracies in the work. And he had reason enough for it. In looking over the work critically with a view of correction, I have been perfectly astonished at the errors that occur on almost every page. And although we understood perfectly what we meant to say, the reader would find it somewhat difficult to decipher our meaning. In the first 140 pages I made nearly 300 corrections. These are practically merely verbal alterations or change of phrases or sentences, so as to make them more accurate or perspicuous. I have in no case so changed the text as to give it a different meaning. I flatter myself that it will now be more worthy the public patronage; and if for no other, this chance for correction I consider alone almost a sufficient remuneration for the brief limits of the copyright. I have also written a preface for the second edition, making quotations from American and European authorities in praise of the merits of the work. From delicacy I have written this as from the publisher. I think it is pretty well done. The work will probably be published in the course of about a month, and those designed for you will be delivered to me, when I shall send them to you. He guarantees not to sell in the State of Missouri or the States south and west of that State. But that, of course, is gammon. The book will be thrown into market and he cannot control the direction in which it will go."—*Jour. Amer. Med. Asso.*

---

### POISONING OF THE UNDERWOOD FAMILY BY WOOD ALCOHOL.*

At 4:30 P.M., February 13, 1899, while one of the worst blizzards of which our New England climate can boast was in progress, I received a summons by telephone from one of our shoe manufacturers that, in a little house in the rear of his factory, a caller had discovered that four of its inmates lay dead on the floor, and others, three in number, were partially unconscious.

In company with the city marshal and one of his force I went immediately to the place and found four dead bodies lying on mattresses on the floor. The bodies, partly dressed, were identified as those of Edward L. Underwood, head of family, aged forty-eight; his daughter, Frances, aged eight years; a daughter Olive, aged twenty-two years. Beside her lay her illegitimate child, John

*Read by E. G. Hoitt, M.D., Medical Examiner, Marlboro, Mass., before the Massachusetts Medico-Legal Society, October 1, 1902.

Clifford, aged two years. The bodies were lying in a natural position; the *rigor mortis* was well developed. All had appearance of having been dead several hours.

I found in an adjoining room Mrs. Adelia Underwood, aged forty-four, wife of Edward, mother and grandmother of deceased children; her son Guy, aged eleven, and one Robert McMullen, not a member of the family but a frequent caller at the place. All seemed in a dazed condition.

Mrs. Underwood made the statement to me that she was afraid that other members of her family in the adjoining room were going to die. She afterwards admitted that she was aware they had been dead since early morning. When asked why she had not notified some one of the fact she said "she did not know."

I asked Robert McMullen his name. He replied "it was none of my business." When asked what he was doing there, said that was his business. I also inquired if he was aware there were dead bodies in the adjoining room. He replied, "What of it?" and "What are you going to do about it?"

When asked if he was acquainted with me, said "no, and did not want to be."

Guy, the boy, stated that none in the house had previously been sick; that all retired the night before upon the mattresses in the following order: First his father, next to him on his left was himself, on his left lay his mother, next Frances, then Olive, and by her side her baby.

He said that McMullen slept on a lounge in the adjoining room. He (Guy) said he awoke about midnight and all seemed to be asleep, the house was warm, saw no smoke and detected no odor. Said his mother soon awoke and gave them all some tea to drink, but was not positive that it was tea, that she also drank of the same, but not from the same cup. Said his mother gave him some tea the night before, which he vomited, and that he did not vomit prior to taking the tea. Said that all of the family drank liquor, and that they gave it to the baby also. He said he awoke at 9 A.M. February 13 and thought his father was dead, and so informed his mother, who made no reply, but went out of the room. He then told McMullen, who came in and looked at them, said he did not believe it. He also went out of the room.

He next learned that little Johnnie, Olive and Frances were dead, and so informed his mother, who attempted to give them some tea, but was too nervous to do so. Said Miss Lillian Clark called in the afternoon to see Olive, and his mother would not let her in, but told her that she had gone to work—said his mother told him to keep still and say nothing.

I ordered the dead bodies to be removed to the city morgue, McMullen to be taken in custody, and placed Mrs. Underwood and her boy Guy under the care of the city physician at police station.

I made an autopsy on Miss Olive in presence of Drs. C. L. Cutler and P. J. Dervin. The body was clothed in chemisette, dress waist and pair of black stockings. There was partial *rigor mortis*, lips and tongue were pale, latter forced against teeth, pupils equally dilated, white froth was oozing from nose and mouth, abdomen large and distended, as though containing foreign body, thumbs flexed on palms of hands, fingers flexed on thumbs. There were no marks of violence on body. The brain was normal in appearance, left lung adherent to diaphragm, otherwise both lungs normal. Pericardium contained about one drachm of yellow serous fluid; heart normal in size and weight; anterior and posterior walls of stomach were congested, also upper part of small intestines. Stomach contained about eight ounces of fluid and some solid particles of undigested food—transverse colon markedly distended with gas and very much congested. Gall bladder contained half an ounce of bile. Liver, kidneys and bladder were normal. Uterus contained eight-pound female child, in eighth month of development.

I will here state that from good authority I learned that two years prior to this circumstance she (Olive) gave birth to a full-term child, which was drowned in a pail of hot water, with her mother's assistance.

I then made autopsy on body of Edward L. Underwood, father of Olive, aged forty-eight years. Body was clothed in black vest, brown flannel outside shirt, light brown undershirt, dark pants, under drawers, one black stocking on left foot, none on right.

There was partial *rigor mortis*, face pale, pupils equally dilated, lips and tongue livid. I found liberally distributed on body pediculi capitis, vestimenti and pubis. Penis was bandaged with white cotton rags, which contained gonorrheal discharge, with some oozing from meatus. Brain was normal in appearance, both lungs normal. Pericardium contained about two drachms of yellow serous fluid. Heart was abnormally large, right auricle, right ventricle and left auricle distended with dark fluid blood, left ventricle empty. Otherwise heart appeared normal. Anterior wall of stomach and piloric end of same, also small intestines, showed inflammation. Stomach contained about six ounces of fluid, no solid particles of food. Bladder distended and contained about one pint of urine. All other abdominal organs appeared normal.

Following this I made autopsy on body of Frances, aged eight years, daughter of Edward. Body was clothed in corset waist, white-ribbed undervest and drawers and black stockings. There was dark fecal matter on nates, seat of drawers and back side of undershirt. Face was pale, eyelids partially open, pupils unequally dilated, lips and tongue livid. *Rigor mortis* was entirely absent. Both pleural cavities contained about four ounces of serous fluid; lungs were normal. Pericardium contained about one ounce of fluid; right auricle distended, right ventricle partially distended with dark fluid blood, left auricle empty, left ventricle fully distended. Heart was normal in size, weight and appearance. Stomach appeared normal, and contained about five ounces of fluid. Small intestines were slightly congested. Bladder contained about one ounce of urine. Liver was normal in size, weight and appearance. All other abdominal organs were normal.

Lastly I made autopsy on body of John Clifford Underwood, aged two years, illegitimate child of Olive, whose autopsy has been previously reported. Body was clothed in dark flannel dress, white undervest and drawers, cotton diaper, containing fecal matter and urine, chest protector, string of Job's tears beads on neck, no shoes nor stockings. Left cheek, nose and forehead were ecchymosed, pupils equally dilated, lips and tongue pale. Each pleural cavity contained about three ounces of amber-colored fluid. All thoracic organs appeared normal. Stomach was distended with gas and slightly congested on anterior surface; contained about four ounces of liquid, no solid food. Gall-bladder contained half a drachm of bile. All other abdominal organs appeared normal. Bladder was empty. After making autopsies on all the bodies I removed from each subject the stomach with contents, portions of intestines, liver and kidneys, and sent same under seal to Dr. Edward S. Wood for his examination.

He reported to me that his analyses of contents of all stomachs revealed wood alcohol in sufficient quantity to produce death.

Later on I learned that it was an established custom of this family to drink anything of an intoxicating nature, and that it was freely given to the younger children. A day or two before their death Miss Olive stole from the factory in which she was employed a quantity of wood alcohol.

Mrs. Underwood, the wife, mother and grandmother, was under the city physician's care for several weeks in a critical condition, but made a good though slow recovery.

Last month she died at the poor-house, from a complication of diseases unknown to the writer. Thus ended this branch of the Underwood family, aside from the boy Guy, now fourteen years of age, who gives promise of following in the footsteps of his degenerate family.—*Boston Medical and Surgical Journal.*

[This description discloses a condition of filth and degeneracy in an old New England town beside which the Georgia negro is as chaste as Niobe and as clean as a lily.—ED. J.-R.]

---

### CONGENITAL DISLOCATION OF THE HIP.*

GENTLEMEN: I shall be as brief as possible in describing the method by which congenital dislocation of the hip may be reduced. For the success of the operation much depends upon the age of the patient. In young children there is generally but little difficulty. In older children and in adults reduction cannot be accomplished. The patients here to-day are from two to nine years of age. In the case of the oldest we may expect to meet with much trouble,

*Delivered at Jefferson College Hospital, Philadelphia, December 11, 1902, by Prof. Adolf Lorenz, of the University of Vienna, Austria. Reported for *The Medical Bulletin.*

for it is near the age-limit, which in most subjects of double dislocation is from the sixth to the seventh year. In unilateral cases this period is extended to the ninth or tenth year. The oldest patient upon whom I have operated was twenty-three years of age, and the result was a success.

In order to effect reduction all the soft parts, especially the long muscles, must be stretched. The adductors offer most resistance. These were formerly incised, but we were liable to divide too much or too little, and there was also the objection of leaving wound-cavites communicating with the air. For these reasons it is better to employ subcutaneous myotomy or to overstretch the structures. The anterior muscles in particular are very refractory. Their opposition must be overcome by traction and, if necessary, by hyperextension. The resistance of the posterior muscles is opposed by flexing the thigh and extending the knee. The next step is to pull down the head of the femur to the acetabulum. Then begins the real process of reduction, the placing of the head of the femur within the acetabulum. This may be done by pressing upon the trochanter, but I prefer to make abduction until I perceive signs of the head of the thigh-bone slipping over the border of the acetabulum. There is, therefore, comparatively little trouble when that cavity is of normal shape and size, as in traumatic cases. In congenital dislocation, however, the acetabulum is too small, and the head of the femur will slip out of the socket. In order to return it to its proper position we must place the limb at a right angle by abduction and also make hyperextension. As regards rotation, it may be made either inward or outward.

It requires no special force to return the head, but the femur must be held in extreme extension. The anterior parts, above all, must be put on the stretch by rotating the thigh. When reduction has been accomplished the contracted flexors of the leg bend the knee. The whole limb must then be stretched while the patient is under the influence of the anesthetic. The limb is kept in a suitable position by being inclosed in a plaster-of-Paris cast, the thigh in the abducted position and the knee flexed, both at right angles.

In unilateral dislocation the patient may be permitted to walk soon after replaciug, but not in bilateral cases. In the latter class the child is kept in bed, and only passive movements are allowed. The cast should be retained rather too long than too short a time. Abundant time must be given. An important factor of success is to keep the head of the femur in place by means of nutritive shrinking of the pelvic muscles, compensated by extreme abduction. This process requires from four to six months. After the muscles have shrunk a less degree of abduction will suffice to maintain the head of the femur in its new position. Sometimes an additional rim of bone will form around the acetabulum. This possibility justifies us in leaving the dressings on for six months, or even for a longer period.

Great care must be taken to retain the cast in good order and in a cleanly condition. It is applied over a stockinet, which reaches from the lower ribs to below the knees, between which and the skin a number of muslin strips or bands extend in every direction. These bands have room enough to move freely beneath the dressing and can consequently be changed. By their means a napkin is held in place to receive the discharges. Both strips and napkins can be changed as often as is necessary, and in this way the cast is kept clean. Before the plaster has set a large opening is cut out around the genitalia and rectum in order to leave plenty of room for the evacuations.

CASE I.—We will begin to-day with the easiest case, that of a female child two years of age. There is dislocation upon both sides. The great trochanters move readily upward and downward. The head of the femur can be felt on both sides. Abduction is limited to a certain extent. The abductor muscles are put upon the stretch. I begin with rupturing their fibers by forced abduction and kneading. The ridge raised by the tendons presently disappears. This manipulation I perform first upon the right and then upon the left side. Next the posterior muscles are put upon the stretch by extreme extension till the child's heel is brought close to the ear. The knee is then flexed and the head of the femur is forced into place. I insert a block under the buttocks, flex the hip, and at the same time make extreme abduction. The acetabulum is poorly developed and must be enlarged by stretching the anterior part of the capsule. The cavity is deeper upon the left side. The X-ray is not of much service in revealing the condition of development of the acetabulum, but this can be ascertained by using the head of the femur as a probe.

After reduction we will keep the thigh bandaged in a position of abduction for six months or more. This is not a difficult case. A second important feature of the after-treatment consists in gymnastic passive exercises for the purpose of obtaining mobility. These movements must be made in such a manner as to keep the head of the thigh-bone in position. They must not be made in the vertical direction of flexion. The movements of flexion and extension must be combined with abduction. Endeavor to exercise the pelvic trochanteric muscles, because they aid in keeping the head of the femur in position. Movements of the knee must also be made in order to avoid contraction. About two years are required to bring the lower limb down to a normal position. The child at first waddles in walking, with its thighs bent outward. Walking helps, by the weight of the body, to keep the femur in the acetabulum.

CASE II.—This is a boy six years of age, and the case is one of the worst which I have encountered. Both hips are involved. The manipulations are the same which I have described and which you have seen me execute.

These children will often creep before attempting to walk. They may aid themselves in the effort to walk by holding in front of them a cane or umbrella.

CASE III.—This girl is nine years of age, and the dislocation is upon the left side. At this age a case is expected to be difficult. The left trochanter is very prominent, a fact which indicates that it is in good condition, but I cannot be sure about the acetabulum. The abductor tendons rise up in hard bands when put upon the stretch, but they at length yield. I now make the usual movements and you can see the head of the bone slip into its socket. In this case I have met with less resistance than I anticipated.

CASE IV.—This little girl, aged four years, has a dislocation upon the left side. Contrary to what one would expect, this case proves to be much more difficult than the preceding. It is, indeed, by far the worst of the series. I shall be exceedingly loath, however, to be foiled in my efforts. The capsule is perhaps too narrow to admit the head of the bone. In this exceptional case I may be compelled to incise the capsule, and if I am driven to that measure it will be the first time in the last 400 or 500 cases. In fact, I have only opened the capsule by the knife three times in more than 1,000 cases. The difficulty here may be due to inflammatory thickening subsequent to tenotomy, which had been performed. Finally, after repeated efforts, I have succeeded in reducing the dislocation. A more complete success could not have been obtained by the open method. It has been necessary to employ extension and counter-extension. Notwithstanding the great force used, there has been no tearing of an artery or other injury to the soft parts. In such young children the tissues are very elastic.

I have entirely abandoned the cutting operation. As regards the results of the bloodless method, we must distinguish between anatomical and functional results. The former are not very good, because the acetabulum was already deformed prior to the operation. The functional result, however, is good. If the head cannot be held in the acetabulum it glides to the surface below the anterior superior spinous process of the ilium, and in that position an up-and-down movement of the thigh is possible. Experience shows that the anatomical result of the open method is also poor in these cases, and much worse functionally, because a secondary contraction of the joint ensues. In the first two of these cases there will be improvement, and this means much to the child. Even if the bloodless method unfortunately fails in certain instances, the condition is, at least, no worse than before the operation.

---

### THE TREATMENT OF PNEUMONIA.

Sir Dyce Duckworth, in the *British Medical Journal* of November 15, 1902, in speaking of the therapy of this disease mentions two symptoms in connection with the nervous system. First of all, as to patients who become delirious at night through exhaustion. That, of course, is more apt to be seen in elderly patients and those who have been intemperate in alcohol. Pneumonia in the drunkard is almost certainly a deadly disease. Double Pneumonia in the drunkard is absolutely fatal; there is no chance for him. Hughes Bennett taught that acute pneumonia in a previously healthy young man or young woman is usually recovered from; but the case is very different if you have an aged patient or one degenerated by abuse of alcohol or any other chronic disease. It should be noted that the average age of the patients of Bennett's favorable statistics was 30$\frac{2}{3}$ years.

Influenzal pneumonia is a very severe form, especially fatal in elderly people. It was so in the last epidemics of influenza. A more important nervous symptom is that of insomnia. The patient cannot sleep, and that, toward the crisis, about the sixth or seventh day, is a very grave matter. It is most important to secure sleep, as the nervous system is exhausted and the patient is worn out with the labored breathing and high fever.

This opens up the debated question as to the use of opium in pneumonia. There are some authorities who maintain that the use of opium in pneumonia is very beneficial. Others aver that the effect of opium is to stop the excretion from the bronchial tubes, to check the power of coughing and the expectoration, and to stupefy the patient to such an extent that he does not feel the need of coughing; a further risk arises from the condition of the kidneys, which may be unsound and unable, therefore, to do an adequate amount of excretory work. It does not do to be too dogmatic, and say that in all cases of pneumonia the use of opium is good or bad. There are some cases in which it is beneficial—those cases in which there is no reason to suppose that the kidneys are involved. A very good plan is to give morphine in small doses with compound spirits of ether; fifteen minims of liquor morphinæ, with one drachm of compound spirits of ether (Hoffmann's anodyne) is an excellent soporific, and will make the majority of these patients sleep. If that is not sufficient, the dose should be repeated. The morphine and ether sustain the action of the heart, and also promote sleep.

The next serious thing in pneumonia that we have to fear, more especially after the age of forty, is failure of heart power. In the majority of cases the heart is unable to stand the strain put upon it by the disease. Where does the strain come from? You can imagine the breathing space in the lung being so diminished that there is comparatively little left. The exudation which fills the air sacs presses upon the small vessels, and so prevents the circulation going on freely, and therefore the blood is thrown back upon the right side of the heart and causes engorgement there. Thence it is thrown back into the venous system, and this becomes engorged. The result is that the patient becomes cyanosed, and sometimes even jaundiced. This is a very dangerous state of matters; not many hearts can bear that strain for long, and if the organ is originally weak, owing to age or degeneration, the strain will prove too much for it and the patient will die. This is a condition which may be relieved by blood-letting; a small bleeding to eight or twelve ounces in such a case may be very useful. Dry pleurisy is a constant accompaniment or part of pneumonia, and you may give relief to the pain of it by the application of two or three leeches.

If the heart begins to falter and the pulse becomes irregular and small—a condition which you may expect in elderly people—you may avert further failure by several measures. First of all you give oxygen. Do not put that off until too late; it is often brought in as the last resource. You had better employ it too soon than too late. If the patient inhales oxygen at intervals for ten minutes at a time that may help him on materially. Again, if the heart begins to fail, you may give great relief by injecting four or eight minims of liquor strychninæ under the skin. This has a marked power of stirring up the cardiac centers, and by this means many people have been saved from death. The author has not found digitalis as useful as strychnine. Now is the time also to use musk. There is only one objection to it, its costliness. We do not use more than a grain or two at a time, but we ought to use more. The old physicians used nine to twelve grains of musk made up in the form of a mixture with syrup and mucilage, and without doubt they got better results than we do with the small doses. When musk is really wanted it is well worth the money. The auther once had evidence of the value of musk in pneumonia from a Sister in John Ward, a most observant and careful nurse. She used to say to the house-physician, "I think the time has come for musk, sir." She had noticed the good effects of it. We generally combine Hoffman's anodyne with tincture of musk and repeat the doses at short intervals.

Of the secondary pneumonia, the author does not say much. The pneumonia which accompanies rheumatic fever he does not think is as severe or as frequent nowadays as it used to be. Treatment by salicylates may perhaps account for this. In this form we find everything classical, except that the patient does not spit up rusty sputa.

Pneumonia may be dependent on gout, and in other conditions may be quite unconnected with the pneumococcus or staphylococcus infections. It is quite certain that pneumonia may occur as a form of visceral gout and replace articular manifestations of the latter.

Note once more that the treatment of pneumonia is strictly the treatment of the patient suffering from it. It is a simple disease in children ; it is not a dangerous disease in the young, or in those who have been previously healthy ; it is more dangerous to those who have reached forty years of age; it is extremely dangerous in the old, and absolutaly fatal in the drunkard.

You might say that the conduct of a case is very simple and demands little more than the practice of common sense. A great deal may be done for this condition by trying to anticipate what may occur. With regard to the use of stimulants there is no routine practice. You should give them in pneumonia just as in other diseases; if they are not wanted, you do not give them. Many cases of pneumonia in the young are best treated without any stimulants at all. But if there be engorgement of the side of the heart, a high temperature, a rapid pulse, the first sound of the heart barely audible at the aortic area (Stokes's indication), stimulants should be given. In the majority of cases two or four ounces of brandy is sufficient in the course of each day. The author has known patients to require even twelve ounces of brandy and they could not have done without it. Your hands are not tied, therefore, in the matter of administering stimulants. It is sometimes more important to give the patient as much pure water to drink as he desires.

This simple matter is too often omitted from the treatment of all febrile conditions.

We may begin our treatment with a mercurial purge, and probably we shall not require any other aperient throughout the case. The subsequent treatment consists of a simple saline, to which some cinchona may be added later on, a diet of milk and beef tea, and above all, watchful and careful nursing. In winter-time successions of hot poultices may be applied to the affected side. In hot weather these may be replaced by cotton-wool sprinkled with spirit of camphor. The author has no liking for a steam-laden atmosphere in cases of pneumonia if the sick-room is properly ventilated. Study the temperature chart, the condition of the heart and anticipate failure of heart power, more particularly in cases where the venous circulation is engorged.—*Therapeutic Gazette.*

---

### An Effective Method of Treating Drug Habits.

Considerable interest has been manifested in the method of treatment outlined below by Dr. Lott, because of the publicity given it by Dr. H. A. Hare of Philadelphia in the *Therapeutic Gazette* and *Medical News.* It has been called the Lott Method from the fact that Dr. M. K. Lott, of Cameron, Texas, called the attention of Dr. Hare to its merits. This gentleman contributes an article upon the subject to the November number of the *Texas Medical Journal.* He states that the treatment is essentially the same as the secret ones advertised by private sanitariums throughout the country, which promise to cure the morphine habit in forty-eight hours. This promise, Dr. Lott shows, is a fallacious one. While the appetite for the drug may be destroyed within this time, the patient is still a man broken in health, his bodily functions impaired and his nervous system and will-power weakened. Many therefore return to the drug unless careful treatment is persisted in to restore the former health.

Not only is the morphine habit treated by this method, but also other drug habituations. In all cases the method is practically the same except that in case of the whiskey habit the bowels should be carefully moved and the patient induced to leave off the stimulant for a day or two before treatment is commenced. For the morphine habit no preparatory treatment is required. The heart and kidneys should be carefully examined, since serious diseases of these organs may contraindicate treatment. If they are crippled but the patient otherwise is in fair condition, strychnia in large doses is used throughout the treatment, which may be continued. Little or no food should be given during the first forty-eight hours, since the digestive processes are suspended and feeding is likely to produce diarrhea. Dr. Lott describes the method as follows:

"The remedies are but few, and most, if not all of them, are quite familiar to the profession. They are in almost daily use. They are: Duboisin, atropia, strychnia and the hydrobromate of hyoscin, hyosciamin, pilocarpin. I have used all of these, but prefer the hydrobromate of hyoscin as the safest and most easily controlled. I usually begin the treatment by securing a quiet, well-ventilated room with but a moderate degree of light, and so arranged that the light can be excluded, for under the influence of hyoscin, or atropin, the pupil is widely dilated, and if there be too much light the eyes are liable to be injured. I secure a careful, competent nurse. He or she should be strong, and able to handle the patient, if necessary, for sometimes, if too much hyoscin be given, the patient will require to be protected against self-injury. The room should be down stairs, preferably. The patient should be given a good bath and put to bed. I give 1-100 gr. of hydrobromate hyoscin, and after this I give the 1-200 gr. from thirty minutes to one hour for from twenty-four to forty-eight hours, until the patient has taken from forty to sixty doses, sometimes decreasing it, and sometimes omitting the dose altogether, watching carefully the pulse and respiration. The object being to secure hyoscin intoxication, which is readily recognized by the restlessness, dilated pupils, dry throat, hallucination, etc., which it produces. When this stage is reached, I give the drug just often enough to keep up the dilirium from twenty-four to forty-eight hours.

"It may be necessary during this period to give a small dose of morphine, even one-fourth grain, once, twice or three times; it will not affect the result in the end.

"Generally it is not necessary during this period to give anything else, though it may be necessary to give strychnia, or nitroglycerin, or digitalis, for the heart; but always remember that hyoscin antidotes morphin, and that morphin antidotes hyoscin. If the patient should get too much of one, I give the other.

"Usually the first few hours, sometimes for four or six hours, but in some instances a much longer period, the patient sleeps. In one instance the patient slept twenty-two hours. The pupils become widely dilated, and the mouth and throat are very dry, and the patient loses the power of coordination and would be unable to stand if permitted to get out of bed; he would likely fall and hurt himself. For this reason, a nurse is constantly at the bedside. Usually after a few hours sleep a delirium comes on and the patient is quite wild. He imagines that he sees and does things that do not occur. If specks are on the quilt, the patient is likely to imagine them to be chinches, and may spend hours picking them off, often remarking on their appearance and number.

"During this period it is necessary to give the patient water frequently, as the throat and mouth are very dry. At the end of this period, generally, the patient is permitted to come from under any drug, and after a few hours reason is restored. The patient expresses himself as cured. He no longer craves the drug, and would not take it.

"At the end of the first stage of treatment, the hyoscin, duboisin, or whichever of the group you have been using, should be left off; and begin a second stage of the treatment with pilocarpin in one-eighth grain doses, which is to be repeated every hour until its physiological effect is secured, then the time between doses is to be lengthened and kept up sufficiently often to maintain the effect, which is elimination of the drugs that have been stored in the cord and in the body; or, in other words, until the last vestige of the symptoms of hyoscin have disappeared. The characteristic sneezing and attempting to yawn now commences.

"During the past forty-eight hours no nourishment, or very little has been taken, and the diarrhea has commenced. This will be likely to vex the patient for some days to come, and until now the urine has been scant and high-colored.

"At this period, the end of forty-eight hours, the patient has been relieved of the morphin, and thus far there has been very little or no suffering. And now pain in the knees, elbows and the back often sets in. Sometimes there are cramps. These are to be relieved, and here the ingenuity of the physician is called into play; for the next few days the real battle is on, and the necessities, real or imaginary, of the patient are to be met.

"For the diarrhea, give subgallate of bismuth in 30 gr. to 60 gr. doses, with fld. extract of coto bark in 20 to 30 drop doses in water at the time of giving the bismuth. This will relieve the diarrhea in a few days. It is at this time the use of pilocarpin $\frac{1}{8}$ gr. and strychnin 1-20 gr. or 1-30 gr., hypodermatically, should be given. Under this treatment the dryness of the mouth and throat passes away, the urine clears up, the skin becomes moist, and the appetite begins to return, and in a week or ten days the stomach will digest almost anything you offer, and the progress is faster. But the patient is nervous and restless. He sleeps poorly, and there are the pains in the limbs and back. For these and the sleeplessness, a bath, either hot or cold, or the Faradic current will be found very beneficial. Under these methods the sleep will soon come naturally. But it may be advantageous sometimes to give large doses of the bromids of potassium, ammonium and sodium a few times, even adding chloral if necessary. But usually it is best to wait for a natural sleep. The pains can be relieved by rubbing with liniments, or by electricity, bathing, pilocarpin, strychnia, atropin, or a few doses of hyoscin.

"Under the stimulus of wholesome, nutritious food, by the end of a week the patient will begin to relish these, and the countenance of the once wretched and wrecked patient, the picture of despair, feeling the warm pulses revive, with richer food and better nourishment, will regain its natural expression and color; and the brightened eye under the power of an invigorated heart, tells of the hope once more kindled in his breast."—*Medical Standard.*

---

**This Journal and Atlanta Semi-weekly Journal one year for $1.25, cash in advance. No checks. Strictly to new subscribers.**

### SALINES IN PERITONITIS.*

Fifty years ago peritonitis was a frequent, dreaded and very fatal disease. A large proportion of the cases were termed idiopathic, which virtually meant that the cause was unknown. We now believe that most of the so-called idiopathic cases were actually cases of appendicitis, which was not fully recognized as a primary disease until many years later. And this is the answer to that conundrum we hear so frequently, "Why are there so many cases of appendicitis now, when the disease was unknown fifty years ago?" Without doubt the disease existed then but was not recognized in its early stage, not diagnosed while limited to the appendical region, and it was only when the inflammation had extended over the abdomen, that it was fairly discovered, but the real cause having been overlooked, the peritonitis was deemed idiopathic or primary. In these days physicians are on the watch, and pain or tenderness in the iliac region is quickly noted, and the suspicion of appendicitis is promptly followed up by every available means for accurate diagnosis and appropriate treatment.

Thus it is that while we have a great number of cases of appendicitis, we have very few cases of idiopathic peritonitis. Appendicitis and puerperal and traumatic peritonitis embrace essentially nearly all the peritonitis met with in these days. The treatment of peritonitis has varied widely, topically, from hot fomentations to the ice-bag and ice-coil; internally through as wide a range.

Watson sums up the treatment prevailing in his time as "blood-letting and mercury." Some writers, even in his day, recommended the use of purgatives, but Watson sets the seal of his disapproval upon them as liable to do harm by increasing peristaltic action. It was deemed advisable to limit or arrest peristaltic action, and to this end opium was recommended. Some already began to realize that bleeding had its limitations, and that under it the asthenic and feeble had their chances of recovery greatly diminished.

The opium treatment in this country was strenuously advocated by Prof. Alonzo Clark, of New York, and that other Dr. Clark of Oswego. It was claimed that intestinal peristalsis was arrested,

*By Benj. Edson, M.D., Brooklyn, N. Y.

pain mitigated and the disease checked. It was claimed that better results followed this method of treatment than that, which it supplanted. But even admitting this, the results were far from encouraging or satisfactory.

It seems almost incredible what enormous doses of opiates were given, and yet some patients recovered, either from the use of it, or in spite of it.

In one of Professor Clark's cases the patient took the equivalent of 1,010 grains of opium in six days, an average of 168 grains a day! The largest dose on any one day was 472 grains on the second day, the smallest 22 grains on the sixth day. We are told that this patient recovered.

At the present time it is pretty generally believed that the *rationale* of the opium treatment was wrong; that it is unwise, if not disastrous, to lock up the secretions, thereby favoring the absorption of septic products conducing to auto-infection, and causing general septicemia. Opiates, if used at all, should be used solely for the relief of excruciating pain.

It is held at the present time that it is far better to eliminate the poisonous products of inflammation and thus give the patient a better chance of recovery. To this end the salines are well adapted. I have relied upon them for many years, and they have met my highest expectations. A case or two may serve to illustrate.

Many years ago I was called up at midnight by a "young man about town" and asked to go over to New York and see a young lady friend of his who was very ill. I reluctantly yielded to his persuasions and went.

The history of the case was that some two weeks before she had an operation performed by a noted abortionist and peritonitis had developed. On reaching the house I found the old reprobate present and thorougly frightened. He had been treating the young woman with opiates by the mouth and hypodermically and she had gone from bad to worse until her condition was pitiable. She had high fever, delirium, tympanites, with abdomen so tense and parts so tender that satisfactory examination was scarcely possible. The vaginal discharge was scanty but malodorous, unmitigated by any cleansing or antiseptic douches. The case was one of those that put the doctor in a quandary as to whether he shall notify the coroner, on the one hand, or on the other, what he shall say in the death certificate.

I decided to use eliminative measures for all they were worth. To that end I ordered magnes. sulph. in half-ounce doses every two hours, to be continued until the patient was thoroughly depleted, every possible ounce of fluid drained away by the bowels. This was done.

I did not visit the patient again—the atmosphere of New York being considered especially uninviting to those having to do with abortion cases—but I directed the treatment through the young man who called at my office daily for instructions. Within a month the young woman herself called at my office, fairly recovered.

The further history of this case is unimportant except as it shows the full recovery as well as the remarkable vitality of this young woman. She is now the wife of this same young man, has borne him three bouncing boys, and boasts of having had seven or eight abortions performed on her by this same old reprobate, and still further avows that she will never bear any more children.

Of course, I do not claim that all my cases have done equally well. Some have been seen only in the last stage of the disease, others have been malignant from the outset. On the whole, however, the salines have proved eminently satisfactory.

Right here it may be asked why this professional abortionist should not be arrested and punished, especially as all the facts of the case are well known and admitted by all the parties concerned.

Well, who is to do it? Who would care to undertake such a thankless and unprofitable prosecution at the expenditure of endless time and money, unpleasant notoriety, and with the almost certain assurance, judging by past experience in such cases, that the guilty would finally escape punishment? Few of us long to become martyrs in such a cause. Most of us have cleaned up cases of this kind, kept our counsel and deemed it prudent to say nothing about them.

I give another case, showing the value of catharsis when needed. A few years ago I was called in consultation to see a young man stopping for the summer at one of our fashionable seaside clubs.

He had been ill for a week or more and under the care of two estimable physicians. Their diagnosis vibrated between appendicitis and typhoid fever. On deep palpation it seemed to me that his bowels were loaded with solid matter. When I suggested a brisk cathartic, I was told that he had already been freely purged. At length, apparently to humor my whim, the attendants consented to give magnesium sulphate a fair trial. The result was all that could be expected—a pailful of feces, with undigested food like clam chowder, green corn, pickled beets, etc., that had been on storage for at least ten or twelve days. Convalescence soon began and typhoid and appendicitis were not in it.

*Revenons a nos moutons.* The use of salines in peritonitis is the rational method of treatment in the great majority of cases.—*Medical Council.*

---

### TETANUS CURED WITH VERATRUM VIRIDE.

An extremely interesting case is reported by Dr. H. B. Sweetser of Minneapolis, in the *Northwestern Lancet* for December 1. The patient was a well-developed girl, 14 years of age, who had been examined several times, per vaginam, to determine the character of a rapidly growing abdominal tumor. She was finally operated upon by Dr. Sweetser, February 27th, and a large ovarian cyst removed; thirteen days later and nineteen days after the first vaginal examination, the first symptoms of tetanus appeared. The disease developed rapidly; it was typical in character, presenting the classical symptoms, including the fixing of the jaws, the risus sardonicus, the clonic convulsions and opisthotonos, and was extremely severe, promising an early and fatal termination. The writer believes that the tetanus bacilli entered the body, not through the abdominal incision, but through abrasions made in the hymen at the time of the earlier vaginal examinations.

The treatment included the use of morphin, chloral, the bromids, paraldehyd and antitetanic serum, but these failing to control the symptoms the author tried the effects of veratrum viride. He says:

"Influenced by the action of veratrum viride in controlling puerperal convulsions, on this fifth day I decided to try it in this case, and at 8 p.m. started timidly with 1 minim doses hourly, but of course without benefit. Looking up its physiological action and dosage I found recorded in Foster's Therapeutics, a case of recovery from tetanus under the use of veratrum viride and gelsemium. This had been reported by Dr. Fordyce Grinnell of Pasadena, Cal., in the *Medical News*, July 18, 1896. The patient was a boy six years old; the period of incubation was nine days; the symptoms fully developed within forty-eight hours; apparently a grave case. Since then I have looked up the literature of tetanus as thoroughly as was possible for me to do in a short time, but curiously have not been able to find a single other case in which veratrum was employed, but did find several cases in which gelsemium was used.

"Therefore on the morning of the 17th I increased the veratrum (Norwood's tincture) to 4 and later to 8 m. and added 8 m. fl. ext. gelsemium, and these were given in combination every hour per rectum.

"This combination controlled the spasms in a remarkable manner, so that by the evening of the same day a very wonderful change for the better had taken place. The clonic spasms became infrequent, but the tonic contractions continued. The pulse became fuller and slower and at times, when she was quiet, went down to seventy-two per minute. As she recovered from the stupefying effects of the chloral she became very noisy and restless. She also vomited a good deal.

"On the 19th the vomiting became so persistent that the doses were reduced to 6 m. every four and then every five hours, and it was decided to return to chloral and bromid, 30 grs. of each per rectum every four hours. Eight doses were given, or ½ oz. of each in all. At the end of twenty-four hours the preceding bad conditions had recurred, and the record showed that the clonic spasms were again being repeated every few minutes and that the pulse was 144 per minute. The chloral and bromid were again stopped, but the veratrum and gelsemium were not increased in frequency until the morning of the 22d, and not in quantity till later on the same day. During the 23d and part of the 24th the veratrum was increased to 10 m. and the gelsemium to 8 m. every hour.

"Again the improvement was marked and prompt, so that she slept nearly all of the night of the 23d and had no spasm after 10.30 p.m. During the 24th the dosage was reduced, still more on the 25th, and stopped on the morning of the 26th. During the 25th, 26th and 27th she had a few slight spasms and four momentary ones on the 28th. There was some question, however, about these, as during these days she was begging for morphin which was being withdrawn.

"In the ten days about 17 drams each of Norwood's tincture and fl. ext. of gelsemium were given per rectum with no effect on the cerebrum and no depressing effect on the system, this being in marked contrast to the action of the chloral."

The symptoms of tetanus, as the author points out, are due to the elaboration of a toxin from the tetanus bacillus of Nicolaier and the presence of this toxin in the blood. The germs are only present at the site of infection and die in ten hours when subjected to the influence of normal tissue. Treatment aims either (1) to neutralize the toxins, or (2) to depress the spinal motor cells until the toxins may be eliminated. The first method of treatment, as represented by antitetanic serum and carbolic acid injections has proven disappointing. There are decided objections to most of the nervous depressants, such as chloroform, chloral and the bromids. Some of these, especially chloral, are decidedly dangerous. Veratrum viride and gelsemium belong in this class and the first one, especially, is (according to Hare) the safest depressant we have, while it undoubtedly acts as an eliminant.—*Medical Standard.*

---

### PARTURITION IN THE VERY YOUNG.

It is natural to suppose that childbirth is fraught with unusual peril to very young women, and we presume that the supposition is very largely entertained in the medical profession. Recorded data on the subject, however, are for the most part little more than fragmentary contributions to its casuistics. Peculiar interest, therefore, attaches to certain observations published by Palotai, of Budapest, in the *Centralblatt für Gynäkologie* for December 27th. He deals only with cases in which the mothers were not over sixteen years old, twenty-five in number. In seven of them the mother was fourteen years old, and in eighteen she had reached the age of fifteen years. Two of the fourteen-year-old girls had never menstruated, and one of the fifteen-year-old ones was said to have been impregnated as the result of rape.

In one of the cases abortion took place in the sixth month of pregnancy; in all the others gestation pursued its course without noteworthy disturbances, though in several it was somewhat abbreviated. In seven instances the precise date of impregnation was accepted as known, and in those cases the average duration of pregnancy was 292 days, rather longer than the usual period. In only two of the mothers, each fifteen years old, were the pelvic dimensions less than normal, and in those there was only slight contraction, and it was limited to the conjugate diameter. How little influence this slight contraction had on the duration of labor is shown by the fact that in one case it was nine hours and twenty minutes, and in the other fifteen hours and nine minutes.

Leaving out of account the case of abortion, which was occasioned by unknown causes, the twenty-four other cases all terminated spontaneously, the child presenting by the vertex. The average duration of labor was ten hours and three minutes; the longest, in a girl fourteen years old, was nineteen hours and thirty-four minutes. The greatest duration of the placental stage was only nineteen minutes. The children were all born alive, and they were no smaller than the average fetus at tern. The largest of them was born of a mother fourteen years old. Although in nearly a third of the cases the vulvovaginal ring was unusually narrow, in only two instances was there sufficient laceration of the peritoneum or of the vaginal wall to require sutures. This goes to show, as has been observed by Staffier, in opposition to the observation of Spitta and Tanner, that youthful primiparæ are not predisposed to perineal laceration. The puerperium did not differ notably from its usual course in older women. Nothing is said of the ability of these young mothers to nurse their offspring. —*N. Y. Med. Jour.*

---

### A NEW CAUSE OF EPILEPSY.

It is stated that the enormous number of cases of epilepsy in the soldiers of South Africa and those who have returned, amounting to over 2,000, were caused not by wounds or any physical injury, but by the harsh screaming detonation of modern firearms, particularly repeating cannon, and by the explosion of Lyddite shells.

